# Current understandings of colibactin regulation

**DOI:** 10.1099/mic.0.001427

**Published:** 2024-02-05

**Authors:** Emily Addington, Sofia Sandalli, Andrew J. Roe

**Affiliations:** ^1^​ School of Immunity and Inflammation, College of Medical, Veterinary and Life Sciences, University of Glasgow, Scotland, UK

**Keywords:** colibactin, regulation, *pks* island, colorectal cancer, genotoxin, virulence

## Abstract

The biosynthetic machinery for the production of colibactin is encoded by 19 genes (*clbA – S*) within the *pks* pathogenicity island harboured by many *E. coli* of the B2-phylogroup. Colibactin is a potent genotoxic metabolite which causes DNA-damage and which has potential roles in microbial competition and fitness of *pks*+ bacteria. Colibactin has also been strongly implicated in the development of colorectal cancer. Given the genotoxicity of colibactin and the metabolic cost of its synthesis, the regulatory system governing the *clb* cluster is accordingly highly complex, and many of the mechanisms remain to be elucidated. In this review we summarise the current understanding of regulation of colibactin biosynthesis by internal molecular components and how these factors are modulated by signals from the external environment.

## Graphical Abstract

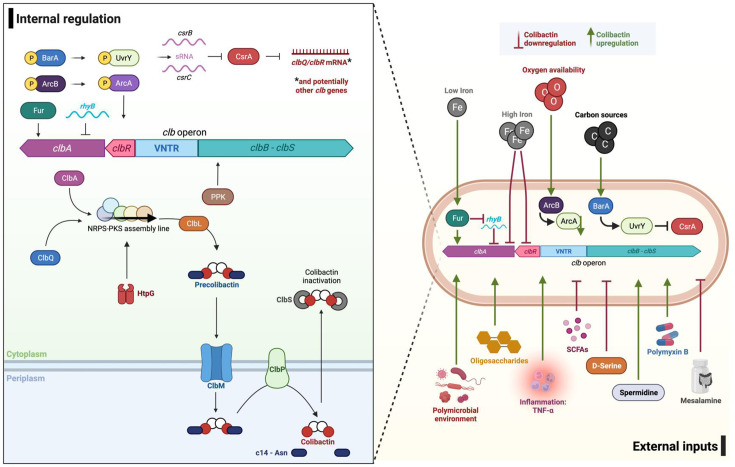

## Introduction

Colorectal cancer (CRC) is the third most commonly diagnosed and second deadliest form of cancer globally [1]. The incidence of CRC is increasing worldwide and is predicted to continue to do so into the future, particularly in economically-impoverished regions where access to screening programmes is limited [2]. Worryingly, there is a rising incidence rate of CRC in young adults, pointing to changes in risk factors during early life [1]. It is well established that there are both environmental (diet, lifestyle) and genetic factors which influence the risk of developing CRC [3]. Individuals with inflammatory bowel disease (IBD) such as ulcerative colitis and Crohn’s disease are at an increased risk of developing CRC, known as colitis-associated colorectal cancer (CA-CRC) [4]. Low-fibre, ultra-processed Westernised diets are known to be linked with increased CRC risk, and the adoption of this diet is likely a factor in the increasing CRC cases being seen in some demographics [1]. Diet heavily influences the composition of the gut microbiome and the role of microbes in CRC causation has become a topic of considerable research, particularly as individuals with CRC often display dysbiosis and greater colonisation with potentially harmful bacteria [5–7].

In 2006, the pathogenicity locus known as the polyketide synthase (*pks*) island was found to encode the biosynthetic machinery for the synthesis of the genotoxin, colibactin. The genotoxicity of this secondary metabolite raised questions as to whether bacteria could have an aetiological role in CRC development [8]. The *pks* island was subsequently shown to be widespread amongst *Enterobacteriaceae*, particularly in *E. coli* belonging to the B2-phylogroup, which are common colonisers of the human intestinal tract [9]. In fact, it is estimated that 20–22 % of healthy individuals are colonised by *pks+ E. coli* [10, 11]. However, *pks*+ bacteria appear to be significantly over-represented in the gut microbiome of patients with CRC, familial adenomatous polyposis (FAP), and IBD, when compared to controls [10–14].

Research following the discovery of the *pks* island has established a strong link between the genotoxin and carcinogenesis, particularly with the identification of a distinct colibactin mutational signature in colorectal carcinomas following exposure to *pks+ E. coli* [15, 16]. Colibactin is a peptide-polyketide genotoxic metabolite and cyclomodulin, capable of inducing DNA damage such as interstrand cross links (ICLs), double strand breaks (DSBs), and single base pair substitutions (SBSs), as well as insertion and deletion (indel) mutations and rearrangement breakpoints. These genomic aberrations may lead to chromosomal instability and cell cycle arrest [8, 17–25].

Characterization of the molecular structure of colibactin led to identification of two electrophilic cyclopropane warheads on opposing sides of the molecule which are capable of alkylating DNA and have high binding affinity for adenine rich motifs (AAAATT), causing DNA cross-linking and activation of DNA repair machinery in human cells [20, 22, 26, 27].

Further to being a potential procarcinogen, colibactin has proposed involvement in bacterial competition, induction of prophages, and manipulation of the gut microbiota composition [28–30]. Indeed, colibactin may have originally evolved as a weapon against competing microbes in the poly-microbial environment, providing advantage to *pks+* bacteria and shaping the surrounding microbial community. Studies in mice indicate that colibactin may exert an effect on the gut microbiota at large, with *pks+* bacteria colonisation resulting in decreased microbial diversity [28]. It has also been shown that colibactin is capable of targeted killing of enteric pathogens and commensals such as *Vibrio cholerae* and *Bacteroides fragilis,* and interspecies culture has been shown to upregulate expression of the *pks* island [29, 31]. Recently, it was demonstrated that colibactin induces lytic development in bacteria containing prophages and that some non-*pks+* bacteria encode the colibactin resistance protein, ClbS [30]. Further, the colibactin intermediary product, N-myristoyl-d-Asn (NMDA) is capable of disrupting the membrane of *S. aureus*, a finding that has led to speculation about the involvement of NMDA in the mechanism of colibactin transport into target cells [31].

Whilst many colibactin-producing strains are pathogenic, certain commensal strains also possess the *pks* island, including Nissle 1917, which has long-standing and proven probiotic effects and is available commercially as a therapeutic.

Despite extensive research into colibactin structure, function, and effect on the human gastrointestinal tract, how the *pks* island and colibactin biosynthesis are regulated remains to be fully clarified. In this review, we aim to provide an up-to-date synopsis of current understanding of regulation of the *pks* island and colibactin determinant.

## The *PKS* island and colibactin biosynthesis

The genomic 54-kilobase *pks* island consists of a total of seven transcriptional units, transcribed as four polycistronic and three monocistronic elements. These are orientated in a single direction, with the exception of a singular contra-orientated polycistron encoding the pantetheinyltransferase (PPTase) (*clbA*) and the LuxR-type transcriptional activator (*clbR*) responsible for promoting downstream *pks* gene expression [8, 32, 33].

The 19 *clb* genes (*clbA – clbS*) of the *pks* island encode the necessary apparatus for the production, transport, and activation of colibactin, including the key polyketide synthases (PKSs), nonribosomal peptide synthases (NRPSs) and hybrid NRPS-PKSs which function as an enzymatic ‘assembly line’ for the genotoxin [8]. Specifically, the biosynthetic cluster encodes three NRPSs (ClbH, ClbJ, ClbN), three PKSs (ClbC, ClbI, ClbO), and two NRPS/PKS hybrids (ClbB, ClbK), as well as accessory and tailoring enzymes (ClbA, ClbD, ClbE, ClbF, ClbL, ClbP, ClbQ, ClbS) with various functions in colibactin synthesis, modification, and self-resistance, or in transport (CbM). The biosynthetic machinery of colibactin is covered in greater depth in other reviews [34–36].

As the colibactin molecule is highly unstable and recalcitrant to isolation, structural predictions and insights into the metabolite have emerged largely from indirect and computational methods. These have revealed that colibactin has a nearly symmetrical structure which is characterised by two electrophilic cyclopropane warheads capable of forming adducts with and alkylating DNA [22, 23, 26, 37–39]. Colibactin biosynthesis involves the sequential addition of building blocks by the enzymatic machinery as the colibactin precursor is transferred between the NRPS and PKS. The metabolite is assembled in an inactive, prodrug form known as ‘precolibactin’ in the bacterial cytoplasm to avoid auto-genotoxicity [37, 40].

Synthesis begins when the PPTase ClbA transfers a phosphopantetheinyl to activate the NRPS and PKS proteins. The NRPS ClbN incorporates asparagine to generate the prodrug motif *N*-myristoyl-d-asparagine (NMDA) which is then transferred to, and accepted by NRPS/PKS hybrid, ClbB. ClbB adds either l-alanine or l-valine to the prodrug, followed by a malonyl-CoA molecule [37].

The prodrug scaffold is then passed along the NRPS-PKS assembly line, to proteins ClbC, -H, -I, -J and -K, undergoing elongation as these enzymes add substrates derived from malonyl-CoA and various l-amino acids such as glycine, cysteine, and methionine [26]. l-Methionine-derived cyclopropane is utilised by ClbH and ClbI, essential for constructing the cyclopropane warheads of the molecule which induce DNA alkylation [27, 39, 41, 42]. Enzymatic studies indicate that a PKS extender aminomalonyl (AM) unit is synthesised by ClbD-F, and is then transferred by ClbG to ClbK, which incorporates it into the precolibactin molecule [39]. The roles of ClbO, ClbL, and ClbQ in the final stages of precolibactin synthesis are still being fully deciphered, and it may be that ClbO is the final enzymatic module of the assembly line and completes the extension stage of the precolibactin skeleton [39]. ClbQ appears to involved in the offloading of compounds from the assembly line [42–44]. Once the prodrug is released from the NRPS-PKS assembly-line, it is reportedly dimerized by the amidase ClbL, the final coupling step in precolibactin biosynthesis [45, 46]. Following dimerization, precolibactin is then transported into the periplasm by ClbM, a multidrug and toxic compound extrusion (MATE) transporter, where it is matured to generate the active genotoxin by the peptidase activity of ClbP, a periplasmic membrane-bound peptidase, which removes the NMDA sidechain. The removal of NMDA induces a spontaneous cyclization cascade resulting in the formation of two electrophilic cyclopropane rings capable of DNA alkylation by ring-opening addition [19, 23, 27, 37, 47, 48]. As the DNA of the synthesising bacteria may be damaged by colibactin should it accidently re-enter the cytoplasm, additional protection from auto-genotoxicity is provided by the hydrolase ClbS, which is able to open and ‘disarm’ the cyclopropane ring on the alkylating warheads [45]. With the exception of *clbS*, activation of all *clb* genes is required for production of mature and genotoxic colibactin [8, 32].

## Internal regulation

### ClbR

Recently, Wallenstein *et al*., (2020) reported that *clbR* encodes the main transcriptional activator of the *pks* operon which regulates colibactin biosynthesis [33]. ClbR appears to be a *pks* island-specific regulator, with ClbR expression directly correlating with functional colibactin production. ClbR itself is characterised as a LuxR-like protein with significant similarity to regulators of the LuxR/FixJ family. The protein possesses a helix-turn-helix DNA binding motif, however, atypically, lacks an N-terminal regulatory receiver (REC) domain. It has been noted that ClbR is highly similar to the GerE transcriptional regulator of *Bacillus subtilis*, which similarly lacks a REC domain, and it has been suggested that both proteins may act as autonomous effector domain regulators [32, 33].

As previously described, the *clb* operon may be considered as consisting of two units. Genes encoding *clbR* and *clbA* are organised in one orientation, whilst the gene cluster encoding for the colibactin biosynthetic machinery, transport, and resistance, is orientated oppositely, beginning with *clbB* and ending with the resistance gene *clbS* [32, 33]. For biosynthesis of the genotoxin to occur, the enzymatic assembly line must be activated, initially with the phosphopantetheinyl transferase, ClbA, which in turn activates the NRPSs and PKSs. Between the two oppositely oriented clusters of the colibactin determinant is an intergenic region comprised of the promoter sequences for the *clbR* and *clbB* genes, as well as variable number tandem repeats (VNTR) of an 8 bp nucleotide sequence, 5′-ACAGATAC-3′, which differs in size amongst *pks+* strains [49]. ClbR appears to interact with this *clbR-*to-*clbB* intergenic region and has a binding site upstream of both *clbB* and its own coding sequence, suggesting that ClbR acts as both a transcriptional regulator and (auto)activator [33]. Located upstream of the *clbR* translational start side, the VNTR region appears to be an additional regulatory unit, affecting *clbR* promoter activity in a manner dependent on the number of repeats in this region. Therefore, VNTR size potentially affects *clbR* transcription, transcript stability, and the efficacy of translation [33].

### High-temperature protein G (HtpG)

The *E. coli* high-temperature protein G (HtpG / Hsp90_EC_) is a heat shock protein and molecular chaperone crucial for colibactin production, and in the absence or mutation of Hsp90_EC_, synthesis of the genotoxin in *pks+E. coli* is abolished [34, 50, 51]. It has recently been shown that direct interaction of Hsp90_EC_ with the *E. coli* chaperone DnaK is required for colibactin production, and results suggest this collaboration with DnaK is necessary for Hsp90_EC_ to correctly fold client proteins [51, 52]. It is possible that Hsp90_EC_ facilitates colibactin production by preventing the misfolding of the colibactin biosynthetic enzymes, a function that would be analogous to what has been shown with the biosynthetic proteins of other PK-NRP compounds and other bacterial Hsp90 molecular chaperones [50, 53, 54]. Current research suggests that the interaction of DnaK and Hsp90_EC_ may serve to protect the colibactin biosynthetic proteins from degradation via the protease HslVU, therefore exerting post-translational control of colibactin production. However, the identity of these client proteins or substrates in the colibactin biosynthetic pathway and how Hsp90_EC_ interacts with these components remains to be clarified [50, 51].

### Polyphosphate kinases

Polyphosphate kinases (PPKs) catalyse the formation of polyphosphate (polyP) from ATP and have vital roles in bacterial functions such as virulence and stress response [55]. Recently, it was shown that mutagenic inactivation of the PPK in *pks+ E. coli* resulted in reduced promoter activity of *clbB* and decreased colibactin synthesis, identifying the PPK as a possible enhancer of colibactin production [56].

The involvement of PPK in colibactin production was further confirmed by use of the anti-inflammatory drug mesalamine, which inhibits PPK enzymatic activity and was shown to decrease *pks*+ genotoxicity [56]. Mesalamine is further covered in later sections of this review.

Notably, PPKs are involved in bacterial stress tolerance, and are activated to synthesise polyP in response to environmental stressors [57, 58]. It is possible that high-stress conditions encountered in inflamed and cancerous tissues may activate the bacterial PPK and enhance colibactin production in these niches. Further research is needed to elucidate the specifics of PPK interactions with the *clb* cluster and involvement in colibactin biosynthesis.

## External inputs

Considering the high metabolic burden of the 54 kb *pks* island and synthesis of a compound as complex as colibactin, it is unsurprising that regulation of this locus appears to be multifactorial, highly complex, and tightly controlled at multiple levels. A coordinated expression of all *clb* genes is necessary for functional colibactin production, and the transcription of this costly biosynthetic machinery would be deleterious to bacterial fitness if expressed under unsuitable conditions [8]. Therefore, the environment of the *pks+* bacterium appears paramount in the regulation of colibactin expression, with external conditions in the bacterial microniche either constraining or promoting colibactin synthesis [35]. To date, various external inputs have been identified which either induce or repress transcription of the colibactin determinant, including carbon source, oxygenation, iron availability, and growth phase [32, 59–61]. Colibactin regulation must respond flexibly to this multitude of varied environmental signals encountered by the bacteria. In the following sections of this review, we will summarise some of the key conditions known to impact regulation of the *pks* island.

### Growth phase and carbon sources

A wide-range of metabolic and virulence-associated traits, including *pks* gene expression, are governed by growth phase of the producing bacterium, as well as by environmental nutrient availability and carbon source. Such resource-dependent and growth-phase dependent regulation is seen with the colibactin operon [32]. Research has shown that promoter activity of the ClbR transcriptional regulator varies during *E. coli* growth, with the highest activity seen at the transition from late exponential phase to early stationary phase in all tested medias, excepting brain heart infusion (BHI) and epithelial cell culture medium, where promoter activity instead peaked at mid-exponential phase [32, 33]. Discussed in greater depth later in this review, this may be orchestrated through the BarA-UvrY system which senses the presence of protonated short-chain carboxylic acids that are produced at the late exponential phase of *E. coli* growth, leading to alterations in colibactin expression [62–64]. Interestingly, it has also been observed that expression of *clb* genes *clbA – clbH* is higher in shaking cultures than in static cultures, but that, conversely, genes *clbJ – clbQ* show significantly lower expression [32].

Nutrient availability demonstrably affects *clbR* promoter activity, with nutrient dense media repressing colibactin expression whilst less complex or minimal media enhances expression [32, 33]. The impact of varying carbon sources has also been investigated and shown to influence colibactin expression. Whilst nutrient dense media typically causes repression of colibactin production, expression of *clb* genes is high in Dulbecco’s Modified Eagle Medium (DMEM), although it remains unclear why this is the case. Specific research into different carbon sources looked at colibactin expression in minimal media supplemented with either glucose, glycerol, pyruvate, or acetate, and reported clear differences in expression of *clb* gene transcription [32]. Subsequent research has since shown the importance of central carbon metabolism on the regulation of colibactin expression, which is discussed in detail later in this review.

To the benefit of bacterial fitness, many *E. coli* virulence factors are induced only upon contact with host cells, and genotoxicity of colibactin in particular requires cell-to-cell contact due to its highly unstable nature and susceptibly to aerobic oxidation [8, 23]. However, a study which examined the expression of various *clb* genes in co-culture with epithelial cells saw no significant induction of these genes upon epithelial cell contact [32].

### Iron availability

Iron bioavailability has been established as a regulator of the *pks* island by directly modulating both *clbR* and *clbA* expression respectively. Iron limitation enhances ClbR-mediated activation of *pks* island transcription, resultingly increasing colibactin synthesis. Similarly, *clbA* transcription is upregulated under low iron conditions and repressed in iron-rich environments [33, 59, 65]. This iron-directed regulation of *clbA* expression appears to be independent of the ClbR transcriptional regulator, instead being positively regulated by the ferric uptake regulator (Fur) protein, and negatively regulated by the small regulatory RNA, *rhyB,* in a non-classical Fur/RhyB pathway [59, 65].

The ClbA PPtase is required for enzymatic activation of the colibactin synthetic machinery and has also been shown to contribute to the production of iron-scavenging siderophores, the biosynthesis of which are regulated by iron availability [66]. Fur, a global transcriptional regulator, is responsible for transcription of over 90 genes involved in iron uptake, storage, and metabolism, and has also been shown to regulate the *stx* genes encoding the Shiga toxin in enterohemorrhagic *E. coli* [66]. The direct binding of Fur to *clbA* positively regulates gene expression, subsequently upregulating colibactin synthesis. Conversely, *rhyB*, a small regulatory noncoding RNA which recruits ribonuclease E (RNase E) to facilitate degradation of mRNA targets, negatively regulates *clbA* transcription through direct binding [59, 67]. It is also possible that *rhyB* may act on colibactin production by repressing expression of serine acetyltransferases, required for incorporating serine as a building block in colibactin synthesis [60, 68].

Interestingly, iron-mediated repression of colibactin synthesis occurs even in a dual *fur* and *rhyB* deletion mutant, indicating that there remains an additional, yet unknown factor involved in iron-directed regulation of the *clb* locus which is Fur/RhyB independent [65]. Such a complex and multifactorial system of regulation likely allows ‘fine-tuning’ of colibactin expression via the interplay of these factors.

The main transcriptional regulator of the *pks* locus, ClbR, is also directly modulated by iron bioavailability, with *clbR* expression enhanced in low iron conditions, thereby increasing colibactin production [33]. Currently, the exact mechanism of iron-dependent regulation of *clbR* is unclear, and no Fur binding sites have been identified upstream of the *clbR* gene [33].

Iron-mediated regulation of colibactin synthesis likely contributes to niche-specificity of *pks+ E. coli* by ensuring that the genotoxin is expressed only in appropriate environments, particularly as iron bioavailability varies widely throughout the body. Many gastrointestinal disorders result in severe iron deficiency, and iron deficiency anaemia is the most common extraintestinal symptom in patients with CRC due to inflammation, poor nutrient absorption, and chronic blood loss [69]. Given the implication of colibactin in the development of CRC, it is worth considering how iron-limited conditions upregulate the expression of this genotoxin and how this may potentially exacerbate tumorigenesis.

### BarA-UvrY and Csr

The carbon storage regulator (Csr) is a global regulatory system known to control multiple bacterial processes, including central carbon metabolism, biofilm formation, quorum sensing, iron storage, and pathogenicity. The Csr system consists of the dimeric mRNA binding protein carbon storage regulator A (CsrA) and the small regulatory RNAs (sRNAs), *csrB* and *csrC*, which are capable of inhibiting CsrA activity [60, 63, 70]. The Csr system is in turn regulated by the BarA/UvrY two-component system (TCS) responsible for modulating adaptive responses in *E. coli*. The BarA (bacterial adaptive response) protein is a sensor kinase, and the UvrY protein is a cognate response regulator of the FixJ family [63]. BarA senses changes in the environment of the bacterium – such as the presence of short chain fatty acids (SCFA) – resultantly activating itself by autophosphorylation and UvrY by transphosphorylation [71]. Activated UvrY is then able to switch-on transcription of the *csrB* and *csrC* sRNAs, which bind to and inhibit CsrA from post-transcriptionally modifying expression of its mRNA targets [63].

Recent work has demonstrated that the BarA-UvrY TCS regulates the *pks* island through mediation of the Csr system [31, 60]. In the absence of an environmental cue, the BarA-UvrY system is inactive and CsrA is free to bind the mRNA of *clb* genes and repress colibactin synthesis. Adding to the complexity of regulation, CsrA-dependent post-transcriptional modulation appears to occur on multiple genes in the colibactin biosynthetic locus. CsrA can bind to *clbR* mRNA encoding the transcriptional regulator of the *pks* island, leading to subsequent downregulation of colibactin synthesis [31]. CsrA has also been shown to bind to the *clbQ* 5′ untranslated leader sequence, repressing expression of the encoded thioesterase, ClbQ, required near the final stages of colibactin synthesis. Additionally, CsrA binding motifs in the upstream region of *clbL* and *clbS*, and in the coding sequence of *clbG, clbI, clbJ*, and *clbN*, have been identified [60]. The binding of CsrA leads to downregulation of the *pks* island and repression of colibactin synthesis.

Alternatively, when the appropriate environmental signals are detected by the BarA sensor kinase, it activates the response regulator UvrY by phosphorylation, which subsequently activates transcription of sRNAs *csrB* and *csrC*. These sRNAs bind to and limit the availability of CsrA, obstructing CsrA from binding to and repressing the *pks* locus [60].

Interestingly, given the proposed role of colibactin in interspecies competition, one of the external signals activating BarA-UvrY-dependent regulation of the *pks* island appears to be a polymicrobial environment [31]. A recent study found that expression of the *pks* island in *E. coli* grown in mixed species macrocolonies was significantly increased compared to that in *E. coli* monoculture macrocolonies, with this increased expression dependent on the BarA-UvrY TCS sensing *S. aureus* and enhancing colibactin expression through inhibition of CsrA [31].

### Oxygen availability

Bacteria which colonise the intestinal mucosa must contend with a steep oxygen gradient along the length of the intestine, as well as anoxia in the intestinal lumen to physiological hypoxia at the epithelial surface [61, 72]. Recent work has shown that oxygen concentration is a direct regulator of colibactin production in *pks+ E. coli*, with genotoxicity optimal in anoxic conditions and repressed in oxygen-rich environments [61]. The colibactin molecule itself is highly-unstable and susceptible to aerobic oxidation and inactivation, thus physical contact between the bacterial and host cell is required for genotoxicity [23]. Given the instability of colibactin and the metabolic expense of its synthesis to the bacterium, it is essential for microbial fitness and pathogenesis that transcription of the colibactin determinant occurs only under viable conditions [61]. That colibactin production is inhibited by oxygen and conversely upregulated in anoxic conditions shows adaption of *pks+ E. coli* to the anoxic intestinal lumen, with oxygen concentration serving as an environmental cue for arrival at a suitable niche.

*E. coli* encode the anoxic redox control (or aerobic respiration control) (Arc) two-component regulatory system consisting of ArcA, a cytosolic transcription factor and response regulator, and the sensor kinase ArcB, responsible for activation of ArcA by transphosphorylation [73]. ArcAB is capable of detecting oxygen availability through quinone levels and correspondingly altering metabolic pathways, and many known pathogenic enterobacteria, including *Shigella,* enterotoxigenic *E. coli*, and uropathogenic *E. coli*, utilise the Arc system to tightly regulate the expression of virulence factors in relation to oxygen concentration [61, 73–76]. Work by Bousset *et al*., (2023) demonstrates that oxygen-induced inhibition of colibactin biosynthesis and genotoxicity occurs via an *arcA*-dependent regulatory mechanism, with anoxic conditions favouring optimal colibactin production. This suggests that *pks+ E. coli* are adapted to anoxic niches such as are found at the surface of the gut epithelium and inside the gut lumen, or to hypoxic niches such as the bottom of the intestinal crypts [61]. It is worth noting that such adaptation would be beneficial for the proposed role of colibactin in bacterial competition within the polymicrobial and anoxic intestinal lumen. Further, hypoxic conditions are a well-established characteristic of malignant tumours, known as tumour hypoxia, with such environments potentially favouring *pks*+ bacteria and optimal colibactin production [77]. Similarly, severe inflammation is associated with local oxygen depletion due to tissue necrosis and the neutrophil respiratory burst, with inflammation also being implicated in promotion of colorectal tumorigenesis by *pks+ E. coli* in murine models [12, 78]. Tissue hypoxia also commonly results from inflammation during bacterial infection [79, 80]. As such, the oxygen-dependent regulation of the colibactin locus via the Arc system helps to ensure that colibactin synthesis occurs in environmental conditions where the genotoxin will be potentized and able to maximise bacterial fitness.

### Inflammation

Inflammation is present in bacterial infections, gastrointestinal disorders, and CRC, and has been shown to promote proliferation and expansion of genotoxic *E. coli* by altering the composition of the intestinal microbiota and by increasing attachment of *pks+* bacteria to an impaired intestinal mucosa [12]. Chronic intestinal inflammation is an established driver of CRC development and is essential for colibactin-induced tumorigenesis, as notably, *pks+ E. coli* fail to produce CRC in inflammation-free murine models [12, 81]. It has been proposed that the carcinogenic microenvironment and the accompanying intestinal inflammation may provide a highly favourable niche for *E. coli,* which are able to utilise inflammation-derived nitrate and formate for growth [82, 83]. Similarly, intestinal inflammation has also been shown to trigger increased oxygen bioavailability in the lumen, providing a fitness advantage for *pks+ E. coli* through aerobic respiration which results in bacterial expansion [83, 84]. Thus, inflammation may enhance colibactin production as a result of increased colonisation of *pks+* bacteria, however, it remains unclear whether inflammation modulates *clb* gene expression directly. RNAseq performed by Arthur *et al.* (2014) indicates that transcription of the *pks* operon is significantly upregulated in response to the inflammatory and carcinogenic environment in mice. Conversely, anti-tumour necrosis factor (TNF) therapy was shown to attenuate CRC development and decrease DNA-damage in mice models infected with colibactin-producing *E. coli* without altering the colonisation of *pks+ E. coli*. This may indicate that the TNF blockade alters *pks* gene expression [85]. In fact, microbial RNA sequencing (RNAseq) performed by the study showed that expression of the *clb* operon was significantly higher when inflammation was present than in controls [85]. This observation is concordant with previous results from Arthur *et al.* (2014) that an inflammatory and carcinogenic environment enhanced *clb* gene activity [81].

Similarly, and of particular note, mesalamine, which is an anti-inflammatory medication frequently used to treat ulcerative colitis, reduces polyphosphate kinase (PPK) enzymatic activity and downregulates colibactin production [56, 86]. Mesalamine is discussed in greater detail in the following section of this review.

Colibactin itself may induce events leading to further inflammation by altering the gut microbiome composition via prophage induction and potential bactericidal activity, and by inducing DNA-damage and cell cycle arrest prompting pro-inflammatory host responses [30, 87, 88]. The relationship between inflammation, colibactin production, and cancer development is therefore highly complex and dynamic, and requires further study to determine and clarify specific mechanisms involved.

### Mesalamine

Mesalamine is an anti-inflammatory bacterial PPK-inhibitor used in the treatment of IBD which has been shown to downregulate expression of *pks* genes and reduce genotoxicity [56, 89]. Recent results indicate that mesalamine reduces colibactin production in both a PPK-dependent and independent manner [56, 90]. Mesalamine acts directly to downregulate colibactin production via inhibition of the bacterial PPK which is involved in synthesis of the active genotoxin. As is discussed earlier in this review, inactivation of the PPK reduces the promoter activity of *clbB*, resultantly diminishing colibactin biosynthesis [56]. The inhibition of PPK activity by mesalamine also serves to decrease *pks+ E. coli* persistence, resistance, and invasion. By decreasing the inflammation which promotes expansion of *pks+* bacteria and colibactin production, and by limiting *pks+ E. coli* colonization and invasion, mesalamine also indirectly affects levels of colibactin synthesis [90, 91]. Mesalamine has also been shown to directly inhibit growth of *pks+ E. coli* in aerobic conditions [89, 92].

### Spermidine

Polyamines are small polycationic molecules produced by all living organisms which have roles in numerous and varied physiological processes, including cell proliferation, stress resistance, and protein synthesis and regulation. As they are required for cell proliferation, polyamines also play a crucial role in carcinogenesis [93, 94]. The predominant polyamines in bacterial cells include putrescine, spermidine, and cadaverine, and these molecules may be synthesised by the bacteria *de novo* or scavenged from the surrounding environment [94]. Spermidine is particularly abundant in cancer tissues, as well as in the gut where it is amply produced by intestinal microbiota [36, 93, 95]. It has been shown that spermidine is involved in colibactin production, and that limited availability of this polyamine results in reduced colibactin synthesis and decreased *pks+ E. coli* genotoxicity [93]. As either spermidine supplementation or limitation affects expression of *clb* genes, and given that polyamines are known regulators of *E. coli* gene expression, it may be that polyamines play a role in monitoring the regulation of colibactin synthesis. Current research suggests that spermidine is capable of positively regulating *clbR* and *clbQ*, however the specific mechanisms and interactions of polyamines in the production of colibactin remain to be elucidated further [36, 93].

### d-Serine

The d-Serine enantiomer is present at varying concentrations within different regions of the human body, and concentration of this amino acid in the gut fluctuates greatly depending on diet. d-Serine can be obtained through dietary sources, such as fermented and fortified foods, or by conversion from l-Serine. Recent work by Hallam *et al*., (2023) has shown that d-Serine downregulates colibactin synthesis and reduces subsequent genotoxicity [96]. Repression of *clb* genes by d-Serine was found to be independent of the serine tolerance locus *dsdCXA* and the exact mechanism of d-Serine-induced *clb* gene repression still remains to be elucidated. It is possible that d-Serine, or a subsequent metabolic product of its catabolism, may act as a cofactor to a transcriptional regulator of the *pks* island [96].

### Other dietary and medical interventions

Numerous other external inputs, including both dietary substrates and medicinal compounds, have been shown to influence expression of the *pks* island and impact colibactin production.

The antibiotic polymyxin B enhances transcription of the colibactin biosynthetic enzymes, thus increasing genotoxin production. It has been proposed that this increase in colibactin synthesis is a stress response to antibiotics, through an unknown mechanism [97].

Oligosaccharides such as inulin and galacto-oligosaccharide (GOS) are prebiotic, and have been shown to upregulate *clb* gene expression and increase colibactin induced genotoxicity in *pks*+ bacteria [98]. Conversely, short chain fatty acids (SCFA) such as acetate, propionate, and butyrate, are produced by fermentation of dietary fibres by gut microbiota and have been shown to downregulate expression of the *pks* island. The mechanism of this process is unknown, although SCFA are established modulators of inflammation [99, 100].

Various plant-derived compounds such as tannin and quercetin have been shown to inhibit growth of *pks+E. coli* and the transcription of *clb* genes [101]. Additionally, one study found that cinnamon and cinnamaldehyde inhibited expression of *clbB* in several *pks+* isolates from CRC patients [102].

### Regulation of colibactin synthesis is complex and occurs at multiple levels

**Fig. 1.**
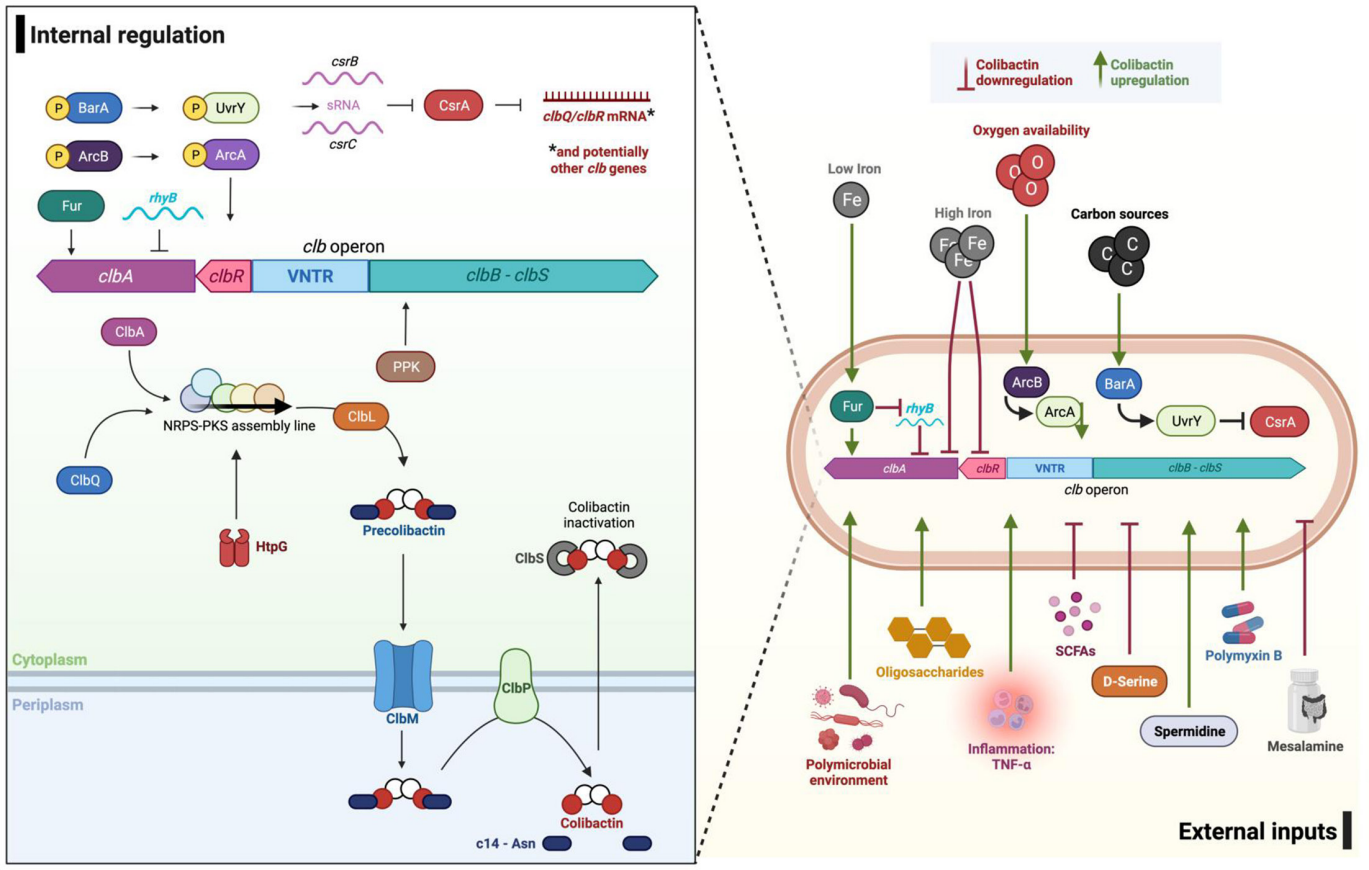
Internal and external inputs effecting regulation of the *clb* genes. (**a**) The 19 genes of the *clb* operon (*clbA – clbS*) encode the biosynthetic machinery required for colibactin production. The *clbR* gene encodes the transcriptional regulator, ClbR, which activates both its own transcription and transcription of the *clb* genes. Level of *clbR* transcription is influenced by the amount of variable number tandem repeats (VNTRs) in the *clbR - clbB* intergenic region, effecting the transcription of other *clb* genes and colibactin production. Colibactin is synthesised in the bacterial cytoplasm as an inactive pro-drug known as ‘precolibactin’ by the enzymatic ‘assembly line’ of polyketide synthases (PKSs), nonribosomal peptide synthases (NRPs) and hybrid NRPS-PKS encoded by the *clb* genes. The *E. coli* high-temperature protein G (HtpG) is also required during biosynthesis. Following dimerization by ClbL, precolibactin is effluxed to the periplasm by the MATE transporter ClbM. The prodrug is then matured into active and genotoxic colibactin by cleavage of the *N*-myristoyl-d-asparagine sidechains by the periplasmic membrane-bound peptidase, ClbP. Should mature colibactin re-enter the bacterial cytoplasm, it is inactivated by the hydrolase ClbS. (**b**) The regulation of the colibactin biosynthetic operon is influenced by a multitude of external environmental factors. Growth phase, nutrient availability, and carbon source impact colibactin expression. Such changes in the external environment are detected by the BarA-UvrY two component system (TCS) which activates the *csrB* and *csrC* small regulatory RNAs which inhibit the carbon storage regulator A (CsrA) from repressing colibactin synthesis. The BarA-UvrY TCS also appears to enhance colibactin expression through recognition of a polymicrobial environment. High oxygen conditions repress colibactin synthesis through the anoxic redox control (Arc) TCS, consisting of the ArcA cytosolic transcription factor and the sensor kinase ArB. Inflammatory environments are advantageous to *pks*+ bacteria and enhance colibactin production, with *clb* gene expression significantly higher when inflammation is present. Conversely, the anti-inflammatory PPK-inhibitor mesalamine downregulates colibactin synthesis, through both PPK-dependent and PPK-independent mechanisms. Anoxic, carcinogenic, and inflamed tissues may therefore be optimal niches for maximal colibactin production. The bioavailability of polyamines, notably spermidine, appears important for colibactin production, with limited spermidine correlating to decreased colibactin synthesis. It is possible that spermidine may positively regulate *clbR* and *clbQ* genes. The metabolite d-Serine has also been shown to downregulate colibactin synthesis, as have short chain fatty acids (SCFA), whilst the antibiotic polymyxin B, and oligosaccharides such as inulin and galacto-oligosaccharides, appear to upregulate colibactin synthesis. Figure by Iris Floria, created with BioRender.com.

Genes encoding for the biosynthesis of colibactin are under strict control at multiple levels to ensure that this metabolically costly machinery is switched-on only under appropriate environmental conditions (Fig. 1). The colibactin operon is transcriptionally regulated by ClbR, a LuxR-type transcriptional regulator, which activates *clb* gene transcription in response to various internal bacterial and external environmental cues. Growth phase, nutrient availability, and carbon source all impact transcription of the *clb* genes and synthesis of colibactin [32, 33]. The carbon storage regulator (Csr) is capable of posttranscriptional modulation of *clb* expression, regulated in turn through the BarA-Uvry TCS which detects changes in the bacterial surroundings [31, 60]. The ferric uptake regulator (Fur), in concert with *rhyB*, also regulates *clb* transcription based on iron availability, downregulating colibactin production in iron-rich conditions and upregulating expression of the genotoxin when iron availability is limited [59, 65]. Similarly, high oxygen conditions decrease colibactin production by inhibition of *clb* transcription which occurs through an Arc-dependent regulatory mechanism [61]. Inflammatory and carcinogenic environments also promote *pks* gene transcription, and anti-inflammatory drugs such as mesalamine have been shown to downregulate transcription of the colibactin biosynthetic machinery [56, 81, 85, 90]. Limited availability of the polyamine spermidine affects expression of *clb* genes, reducing colibactin synthesis through an unknown mechanism [93]. Similarly, the heat shock protein Hsp90_EC_ is required for colibactin production, and may act as a chaperone for the enzymes comprising the colibactin biosynthetic machinery [50]. Diet is known to have profound consequences on the microbiota and environment of the intestinal tract, and many dietary modulators of colibactin gene expression have been identified, including oligosaccharides such as inulin and short chain fatty acids produced by dietary fibres [98, 99]. d-Serine has also been shown to downregulate transcription of *clb* genes and repress colibactin synthesis [96].

It is apparent that *pks*+bacteria tightly regulate the production of colibactin through a number of mechanisms, in a highly specific manner dependent on numerous environmental cues that signal the presence of either optimal or deleterious conditions. Such regulatory systems maximise fitness of the bacterium, minimising unnecessary metabolic burden and facilitating appropriate production of the metabolite to the benefit of bacterial competition, survival, and pathogenesis.
